# Hypofractionated palliative volumetric modulated arc radiotherapy with the Radiation Oncology Study Group 8502 “QUAD shot” regimen for incurable head and neck cancer

**DOI:** 10.1186/s13014-020-01548-w

**Published:** 2020-05-27

**Authors:** Ryo Toya, Tetsuo Saito, Kohsei Yamaguchi, Tomohiko Matsuyama, Takahiro Watakabe, Tadashi Matsumoto, Ryoji Yoshida, Akiyuki Hirosue, Daizo Murakami, Yorihisa Orita, Hideki Nakayama, Natsuo Oya

**Affiliations:** 1 Department of Radiation Oncology, Faculty of Life Sciences, Kumamoto University, 1-1-1 Honjo, Chuo-ku, Kumamoto, 860-8556 Japan; 2 Department of Oral and Maxillofacial Surgery, Faculty of Life Sciences, Kumamoto University, 1-1-1 Honjo, Chuo-ku, Kumamoto, 860-8556 Japan; 3 Department of Otolaryngology-Head and Neck Surgery, Faculty of Life Sciences, Kumamoto University, 1-1-1 Honjo, Chuo-ku, Kumamoto, 860-8556 Japan

**Keywords:** Head and neck cancer, Radiotherapy, QUAD shot, Volumetric modulated arc therapy, Intensity-modulated radiotherapy, Hypofractionated radiotherapy, Palliative treatment

## Abstract

**Background:**

To review a single institutional experience of the Radiation Therapy Oncology Group (RTOG) 8502 “QUAD shot” regimen using volumetric modulated arc radiotherapy (VMAT) for incurable head and neck cancer (HNC).

**Methods:**

Thirty-four consecutive patients with HNC were treated with at least one cycle of the RTOG 8502 regimen. Treatment plans included the use of VMAT with 6 MV photons generated by a linear accelerator. Two daily fractions of 3.7 Gy were delivered with an interval of at least 6 h for 2 consecutive days, totaling 14.8 Gy over 4 fractions. This was repeated every 3–4 weeks for a total of three cycles. No concurrent systemic therapy was performed.

**Results:**

The number of completed cycles was 1 in 6 (18%) patients, 2 in 5 (15%), and 3 in 23 (68%). Tumor response was achieved in 29 (85%) patients and symptom relief in 20 (77%) of 26 patients. Overall response (tumor response or symptom relief) was achieved in 32 (94%) patients. All patients who received 2 or more treatment cycles achieved overall response. Median overall survival (OS) was 5.7 months. Multivariate analysis revealed that completion of all three treatment cycles was significantly associated with better OS (*P* = 0.002). Grade 2 toxicity was observed in four (12%) patients, but no acute Grade ≥ 3 or late toxicity was observed.

**Conclusions:**

The RTOG 8502 “QUAD shot” regimen using VMAT is effective for incurable HNC with highly reduced toxicity. Treatment with multiple cycles is recommended for better treatment response and/or survival.

## Background

Patients with head and neck cancer (HNC) are often ineligible for curative therapy such as surgery and definitive radiotherapy (RT) because of advanced age, poor performance status, extent of the tumor, prior treatment, and comorbidities. Patients with HNC often have symptoms such as pain, hemorrhage, dysphagia, and airway compromise which decrease the quality of life (QOL) [1, 2].

In the 1980s, the Radiation Therapy Oncology Group (RTOG) performed a phase II study of RT, which consisted of 2 days of twice-daily fractionation with a fraction size of 3.7 Gy (14.8 Gy per cycle) repeated at 3 to 6 week intervals for a total of three cycles with an RT dose of 44.4 Gy for pelvic malignancies [3]. Thereafter, this RTOG 8502 “QUAD shot” regimen has been successfully adapted for palliative treatment of HNC. The RTOG 8502 regimen for HNC has been reported to achieve tumor response and palliation in approximate 50 to 85% and 55 to 100% of patients, respectively. Furthermore, toxicity has been reported to be mild, with Grade 3 toxicity present in approximately 0–10% of patients [2, 4–8]. The current National Comprehensive Cancer Network (NCCN) guidelines recommend the RTOG 8502 regimen using three-dimensional conformal RT (3D-CRT) or intensity-modulated radiotherapy (IMRT) as one of the palliative RT regimens for HNC [9].

Recently, volumetric modulated arc therapy (VMAT) has been introduced to treat HNC [10]. Using this technique, the gantry is rotated while the dose is being delivered, and three parameters (dose rate, field shape, and speed of gantry rotation) can be changed as the beam is rotated [11]. Compared with conventional fixed-field IMRT, VMAT provides similar excellent dose coverage to the target volume with a reduced dose to organs at risk (OAR). Furthermore, the treatment time of VMAT is much shorter than conventional IMRT; the approximate treatment times are 2 to 4 and 10 to 15 min for VMAT and IMRT, respectively [10]. Introduction of VMAT into palliative RT regimen with RTOG 8502 may provide good treatment response with reduced toxicity for patients with HNC. To the best of our knowledge, the treatment results of RTOG 8502 regimen using VMAT has not been evaluated. The purpose of this study was to review a single institutional experience of the RTOG 8502 “QUAD shot” regimen using VMAT for incurable HNC.

## Methods

### Patients

This retrospective study was approved by the institutional review board of our hospital. Prior informed consent for treatment was obtained from all patients. Between January 2018 and July 2019, 34 consecutive patients with HNC were treated with at least one cycle of palliative RT with the RTOG 8502 regimen. Eligible patients had histologically or cytologically proven malignancy from primary of head and neck origin or large nodal metastasis from an unknown primary suspected to be of head and neck origin. They were ineligible for definitive or systemic therapy due to disease extent, extensive comorbidity, or refusal to undergo conventional treatment; hence, no patients received concurrent systemic therapy during treatment. The minimum interval between prior RT and re-irradiation with the RTOG 8502 regimen had to be 6 months.

### Radiotherapy details and technique

Patients were simulated with planning computed tomography (CT) imaging in a dedicated thermoplastic head and neck mask for immobilization prior to each RT cycle. Gross tumor volume (GTV) included primary tumor and lymph node metastases; it is defined based on the fiber scope, contrast-enhanced CT images, and magnetic resonance (MR) imaging with or without [^18^F]-fluoro-2-deoxy-D-glucose (FDG)-positron emission tomography (PET)/CT [12, 13]. A clinical target volume margin of 5 mm was added to the GTV for subclinical invasion. Planning target volume (PTV) margins of 3 mm were added to cover setup errors. Treatment plans included the use of VMAT (RapidArc; Varian Medical Systems, Palo Alto, CA, USA) with 6 MV photons generated by a linear accelerator (Clinac iX; Varian Medical Systems, Palo Alto, CA, USA). The plan was generated using 1 arc rotating from 181° to 179° clockwise with the dose rate varied between 0 MU/min and 600 MU/min.

RT was delivered using the RTOG 8502 “QUAD shot” regimen. Two daily fractions of 3.7 Gy were delivered with an interval of at least 6 h for 2 consecutive days, totaling 14.8 Gy over 4 fractions. This was repeated every 3–4 weeks for a total of three cycles and a total dose of 44.4 Gy. The goals of the VMAT plans for target volume were defined as follows: dose to 95% of the PTV (D_95_) > 95% of the prescribed dose, the percentage of the PTV receiving 93% of the prescribed dose (V_93%_) > 99%, the percentage of the PTV receiving 110% of the prescribed dose (V_110%_) < 1%. The maximum allowable dose limit of the spinal cord and brainstem was defined as a total dose of 30 Gy and 50 Gy for the patients without and with prior RT, respectively. The mean dose of each treatment cycle was <5Gy for at least one parotid gland. The dose to the mucous membrane and skin, which were non-adjacent to the target volume was reduced as little as possible. The cone-beam CT (CBCT) scans were acquired using a kilovoltage CBCT scanner and images of CBCT were registered to planning CT for image guidance of each treatment session. For patients with two or more treatment cycles, adaptive radiotherapy (ART) was performed to adjust to their anatomic changes and to avoid overdosing of normal tissues and underdosing or marginal geographic misses of target volumes during a course of treatment by repeating planning CT imaging and replanning for every cycle.

### Evaluation of treatment response and toxicity

Tumor response, symptom relief, and toxicity were assessed every 2 weeks until 1 month after the final course of treatment, and every 3 to 4 weeks thereafter until patients died or were no longer able to comply [5]. Tumor response was evaluated by physical examination and/or radiographic tumor response, and defined as objective shrinkage of GTV [2, 6]. Symptom relief was defined as subjective reduction of the presenting symptom(s) [2, 8]. Overall response was defined as tumor response or symptom relief. Toxicity was scored by the Common Terminology Criteria for Adverse Events version 5.0. Acute toxicity was defined as occurring up to 3 months after treatment completion [2].

### Statistical analysis

Overall survival (OS) and progression-free survival (PFS) rates were calculated from initiation of RT using the Kaplan-Meier method. Candidate variables for prognostic factors of OS and PFS, including age, Eastern Cooperative Oncology Group performance status, tumor site, histology, clinical stage, prior RT at the palliative site, and treatment cycles were evaluated by univariate analysis using log-rank statistics. To determine the independent significance of variables, multivariate analyses were performed using the Cox proportional hazards model, by selecting significant variables on univariate analysis. Differences with *P*-values < 0.05 were considered statistically significant. Statistical calculations were performed using SPSS software, version 26.0 (SPSS Inc., Chicago, IL, USA).

## Results

### Patient and treatment characteristics

Patient characteristics are summarized in Table 1. The majority of patients were 75 years or older (*N* = 25, 74%). The histology was squamous cell carcinoma in 28 (82%) patients, salivary duct carcinoma in 2 (6%), intestinal-type adenocarcinoma in 1 (3%), papillary carcinoma in 1 (3%), verrucous carcinoma in 1 (3%), and poorly differentiated carcinoma in 1 (3%). Seven (21%) patients had received prior RT at the palliative sites. The number of completed cycles was 1 in 6 (18%) patients, 2 in 5 (15%), and 3 in 23 (68%). Eleven (32%) patients discontinued treatment because of the reduced performance status (*N* = 4), refusal of treatment (*N* = 3), pneumonia (N = 2), and progression of coexisting hepatocellular carcinoma (*N* = 1) and dementia (*N* = 1).

**Table 1:** Patient characteristics (*N* = 34)

Characteristics	*N*	(%)
Age (years)	Median 81 (range: 54–92)	
Gender		
Male	18	53
Female	16	47
ECOG performance status		
0	5	15
1	8	24
2	11	32
3	10	29
Tumor site		
Oral cavity	19	56
Nasal cavity and paranasal sinuses	5	15
Hypopharynx	4	12
Skin	2	6
Major salivary gland	2	6
Thyroid	1	3
Neck disease with an unknown primary	1	3
Histology		
Squamous cell carcinoma	28	82
Others	6	18
Clinical stage		
II	3	9
III	3	9
IVA	17	50
IVB	9	26
IVC	2	6

Abbreviations: *ECOG* Eastern Cooperative Oncology Group

### Treatment response

Tumor response was achieved in 29 (85%) patients (Table 2). Twenty-six (76%) patients had pretreatment symptoms; the predominant presenting symptoms were pain (*N* = 24, 71%) and hemorrhage (*N* = 9, 26%). Symptom relief was achieved in 20 (77%) of the 26 patients. Overall response was achieved in 32 (94%) patients.

**Table 2:** Tumor response, overall response, and toxicity of Radiation Therapy Oncology Group 8502 regimen

Completed cycles	*N*	Response				Toxicity			
		Tumor response		Overall response		Grade 1		Grade 2	
		*N*	%	*N*	%	*N*	%	*N*	%
1	6	3	50	4	67	1	3	0	0
2	5	5	100	5	100	1	3	0	0
3	23	21	91	23	100	7	21	4	12
Total	34	29	85	32	94	9	26	4	12

### Survival

**Fig. 1:**
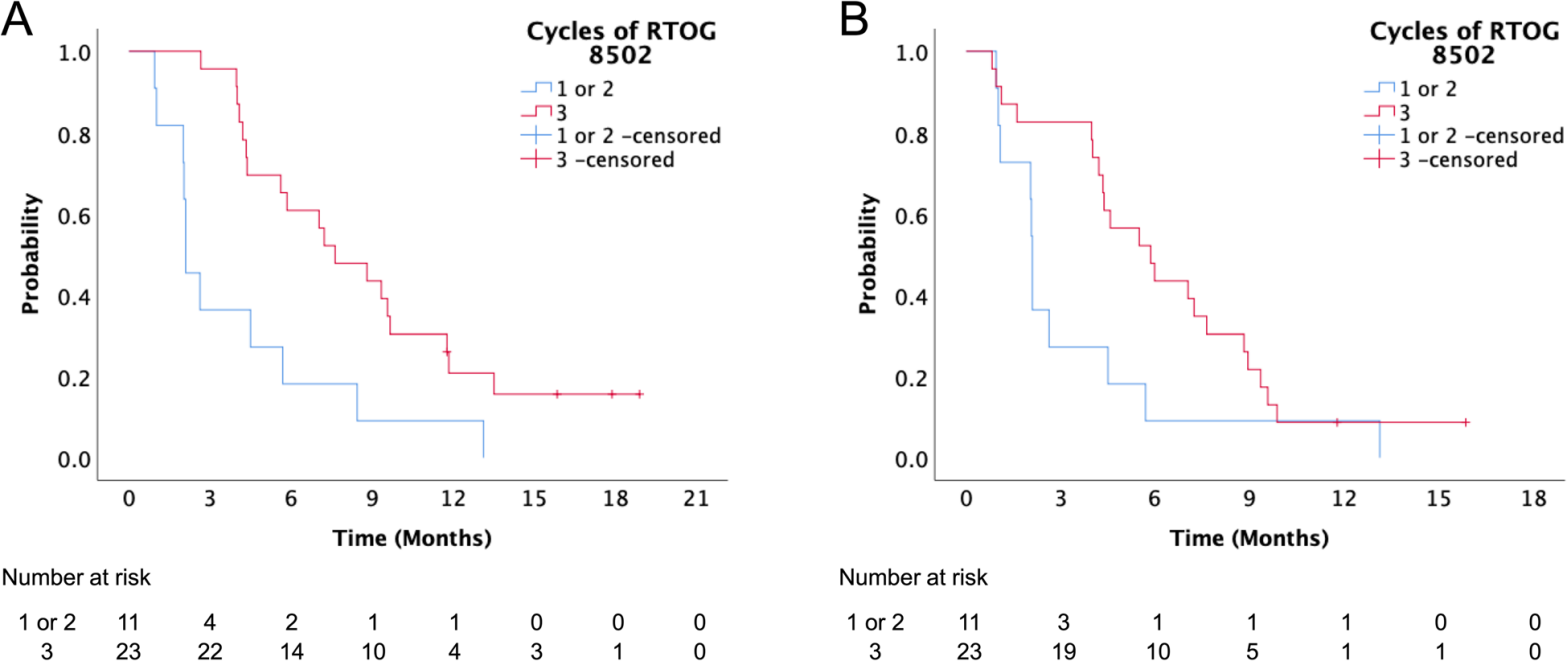
Overall (**A**) and progression-free (**B**) survival curves according to completed cycles of Radiation Therapy Oncology Group (RTOG) 8502 regimen

At the time of analysis, 32 (94%) patients had died. The median follow-up duration was 5.8 (range: 1.0–18.9) months. Median OS was 5.7 (range: 1.0–18.9) months. Univariate analysis showed that clinical stage II-III (*P* = 0.046) and the completion of all three treatment cycles (*P* = 0.003) were significantly associated with better OS (Table 3, Fig. 1). These two factors remained as independent variables for OS in a multivariate analysis (*P* = 0.023 and *P* = 0.002, respectively). Median PFS was 4.4 (range: 0.8–15.9) months. The univariate analysis revealed that only the completion of all three treatment cycles was significantly associated with better PFS (*P* = 0.045, Table 3, Fig. 1).

**Table 3:** Univariate and multivariate analysis of the overall and progression-free survival

Variable	N	OS			PFS
		UVA	MVA		UVA
		*P*-value	HR (95% CI)	*P*-value	*P*-value
Age (years)					
< 80	12	0.684	NA		0.265
≥ 80	22				
ECOG performance status					
0–1	13	0.375	NA		0.847
2–3	21				
Tumor site					
Oral cavity	19	0.627	NA		0.647
Others	15				
Histology					
SCC	28	0.886	NA		0.482
Others	6				
Clinical stage					
II-III	6	0.046	1	0.023	0.321
IV	28		1.922 (1.096–3.371)		
Prior RT at the palliative site					
Yes	7	0.910	NA		0.776
No	27				
Cycles of RTOG 8502					
1–2	11	0.003	3.711 (1.652–8.340)	0.002	0.045
3	23		1		

Abbreviations: *ECOG* Eastern Cooperative Oncology Group; *SCC* squamous cell carcinoma; *RT* radiotherapy; *RTOG* Radiation Therapy Oncology Group; *OS* overall survival; *PFS* progression-free survival; *UVA* univariate analysis; *MVA* multivariate analysis; *HR* hazard ratio; *CI* confidence interval; *NA* not applicable

### Toxicity

Overall, Grade 1 acute toxicity was observed in nine (26%) patients, with the most common being mucositis (*N* = 7) and dry mouth (*N* = 3). Grade 2 acute toxicity was observed in four (12%) patients and consisted of mucositis (*N* = 4) and dry mouth (*N* = 1) (Table 2). No acute Grade ≥ 3 or late toxicity was observed. Of the seven patients who had received prior RT at the palliative sites, Grade 1 acute toxicity was observed in one (14%) patient with dry mouth. Grade 2 acute toxicity was observed in two (26%) patients and consisted of mucositis (*N* = 2) and dry mouth (*N* = 1).

## Discussion

RT for incurable HNC has been demonstrated to be an effective palliative modality, even for patients who have received prior RT [2, 14]. Currently, no consensus exists for appropriate palliative RT regimen in HNC. In general, a once-daily hypofractionated RT regimen of 30 Gy/10 fractions is commonly performed as palliative RT regardless of the tumor site; however, this treatment regimen is inappropriate for HNC because of the acute adverse effects. The reported frequency of ≥Grade 3 acute toxicity with this treatment regimen for patients with HNC was > 40% [6]. Other hypofractionated palliative RT regimens have been reported for HNC. Stevens et al. performed palliative RT for 148 patients with newly diagnosed HNC [15]. The median RT dose and fraction number were 50 Gy and 20, respectively; the most frequently used fractionation regimen was a split course designed to deliver a total dose of 50 Gy in 2.5-Gy fractions within 6 weeks, composed of two cycles of 25 Gy in 10 fractions given within 2 weeks, separated by a 2-week break. Overall response was reported in 85 (57%) patients, while 10 (7%) and 8 (5%) patients had unplanned discontinuation and planned RT interruption because of toxicity, respectively. The “Hypo Trial” conducted by Porceddu et al. treated 35 incurable patients with HNC; patients received 30 Gy in five fractions at 2/week, at least 3 days apart, with an additional boost of 6 Gy for small volume disease (≤ 3 cm) in suitable patients [16]. Tumor response was achieved in 28 (80%) patients. Grade 2 and 3 mucositis were reported in 13 (37%) and 9 (26%) patients, respectively, and Grade 2, 3 and 4 dysphagias were reported in 23 (66%), 4 (11%) and 2 (6%) patients, respectively. These RT regimens provide certain palliative response; however, acute adverse effects that may decrease patients’ QOL are still relatively strong. Palliative RT should be considered for relief or prevention of locoregional symptoms; however, severe toxicity should be avoided [9]. Our results suggest that RTOG 8502 regimen using VMAT is one of the strongest candidates of palliative RT regimens with good treatment response and low toxicity.

In earlier studies, RTOG 8502 regimen was performed using a two-dimensional (2D) RT. RT field was typically defined as the gross symptomatic disease plus a 1–2 cm margin based on physical examination [4, 5]. Nowadays, modern diagnostic imaging of MR and FDG/PET-CT, which achieve precise target definition and reduced target volume, has been included in RT planning [12, 17–19]. Furthermore, the technical development of RT techniques in the last 2 decades, such as 3D-CRT, IMRT, VMAT, ART, and image-guided RT (IGRT) based on CT images, provides an enhanced dose concentration to the target volumes, reduces dose to OARs, and promises precise RT delivery [11, 20–24]. These sophisticated treatment techniques are of significant value for not only definitive RT but also palliative RT with regard to treatment response and toxicity.

The clinical outcome of RT with RTOG 8502 regimen for patients with head and neck tumors is summarized in Table 4. Paris et al. reported the results of phase I-II study of RTOG 8502 regimen without chemotherapy for incurable HNC in 1992 [4]. They treated 37 patients with 39 lesions with 2D-RT technique using Cobalt 60 or 6 MV photons. The spinal cord dose was limited to 30 Gy by field reduction. Twenty-one (57%) patients completed all three cycles and tumor response was achieved in 30 (77%) of the 39 treated lesions. A decade later, Corry et al. reported the results of phase II study of palliative RT with a similar QUAD shot regimen without chemotherapy for incurable HNC [5]. They performed RT to a maximum of three cycles with a fraction size of 3.5 Gy, which differed slightly from the original RTOG 8502 regimen. Radiation was delivered using 2D-RT and the spinal cord was excluded to limit its dose to 28 Gy in 8 fractions. Sixteen (53%) patients completed all three cycles and 16 (53%) patients achieved a tumor response. Our tumor response results were superior to those achieved with 2D-RT. One of the reasons for this superiority may be that dose coverage for the target volume using VMAT is superior to that of 2D-RT because it is technically difficult to provide uniform distribution to the target volume using 2D-RT while reducing spinal cord dose within the limitation. Recently, Gamez et al. reported treatement results of RTOG 8502 regimen in stage III–IV head and neck tumors [8]. All 21 patients underwent concurrent systemic therapy: 18 (86%) and 3 (14%) patients received carboplatin and cetuximab, respectively. Radiation was delivered using a 3D-CRT in 6 (29%) patients and IMRT in 15 (71%) patients. Sixteen (76%) patients completed all three cycles and 18 (86%) patients achieved a tumor response. Although we did not perform concurrent systemic therapy, our tumor response of VMAT alone was similar to theirs.

**Table 4:** Treatment outcomes of the Radiation Therapy Oncology Group 8502 regimen for head and neck cancer

Author	Year	Design	N	Population	SCC(%)	Age (years)	Systemic therapy (%)	RT technique			Treatment cycle (%)					Outcome					Toxicity (%)	
								2D-RT	3D-CRT	IMRT	1	2	3	4	5	TR (%)	SR (%)	OR (%)	OS (months)	PFS (months)	G2	G3
Paris [4]	1993	Phase I–II	37	Incurable H&N tumors	79	66	0	100	0	0	43^c^		57	0	0	77	85	NA	4.5	NA	NA	NA
Corry^a^ [5]	2005	Phase II	30	Incurable HNSCC	100	73	0	100	0	0	20	27	53	0	0	53	56	NA	5.7	3.1	37^d^	0
Chen [6]	2008	R	23^b^	Metastatic HNSCC	100	70	0	20	65	15	26^c^		74	0	0	NA	NA	83	4	NA	NA	9
Lok [2]	2015	R	75	Incurable H&N tumors	55	76	29	0	45	55	36	27	32	4	1	NA	NA	65	5.7	NA	28	5
Finnegan [7]	2016	R	70	Stage III–IV HNSCC	100	66	56	0	49	51	24^c^		76	0	0	NA	61	NA	3.9	3.4	20^e^	6
Gamez [8]	2017	R	21	Stage III–IV H&N tumors	NA	62	100	0	29	71	0	24	76	0	0	86	100	NA	7	4	35	0
Our study		R	34	Incurable H&N tumors	82	81	0	0	0	100	18	15	68	0	0	85	77	94	5.7	4.4	12	0

Abbreviations: *R* retrospective study; *H&N* head and neck; *HNSCC* head and neck squamous cell carcinoma; *SCC* squamous cell carcinoma; *RT* radiotherapy; *2D-RT* two-dimensional radiotherapy; *3D-CRT* three-dimensional conformal radiotherapy; *IMRT* intensity-modulated radiotherapy; *TR* tumor response; *SR* symptom relief; *OR* overall response; *OS* overall survival; *PFS* progression-free survival; *G* Grade

^a^Corry et al. used a fraction size of 3.5 Gy

^b^Subset of 60 patients

^c^Treatment cycles of 1-2

^d^Salivary gland toxicity

^e^Mucositis

The previous reported symptom response and overall response of the RTOG 8502 regimen with or without systemic therapy approximated 55–100% and 65–85%, respectively [4, 5, 7, 8] (Table 4). Our results of symptom response were comparable to these results. Furtheremore, our results of overall response were superior to these results. RTOG 8502 regimen using VMAT provides appropriate treatment response without using systemic therapy: this treament strategy may improve or maintain patient QOL.

The previously reported median OS and PFS of the RTOG 8502 regimen with or without systemic therapy approximated 4–7 months and 3–4 months, respectively [4, 5, 7, 8] (Table 4). Our results were similar with these results. Considering that the prognosis of patients with HNC who undergo non-curative treatment is poor with approximate survival time of 2–4 months [25, 26], palliative RT may contribute to a certin degree of prolonged survival. However, more than half of the patients die within 6 months even if they undergo palliative RT, including RTOG 8502 regimen [4, 5, 7, 8, 15, 16]. Therefore, a smaller number of fractions such as RTOG 8502 regimen is feasible for the palliative RT.

In our series, all patients who received 2 or more treatment cycles achieved overall response. Furthermore, completion of all three treatment cycles is significantly associated with better OS and PFS, which is consistent with previous reports [2, 8]. Treatment with multiple cycles is recommended for better treatment response and/or survival.

The incidence of the Grade 2 and 3 toxicities in patients with HNC treated with RTOG 8502 regimen was reported as approximately 20–40% and 0–10%, respectively [2, 5, 7, 8] (Table 4). Our results of toxicities were much lower than those of previous reports. Furthermore, toxicity was acceptable even in the patients who had received prior RT at the palliative sites. The primary reason for highly reduced toxicity in our patients may be attributed to the use of VMAT, ART, and IGRT. Another reason is that we did not perform concurrent systemic therapy. We recommend the introduction of these sophisticated treatement techniques into palliative RT regimen with RTOG 8502 not only because of their excellent palliative response but because of the highly reduced toxicity.

However, this was a retrospective study based on a relatively small number of patients. The potential for selection bias exists, which may influence the results of the treatment outcomes and analysis. Similarly, we could not evaluate the influence of palliative RT on overall QOL of the patients because our study was retrospective. A prospective trial with a larger cohort should be performed to further evaluate the value of introducing modern sophisticated RT techniques into RTOG 8502 regimen for HNC patients. Further investigations are underway to assess this concern.

## Conclusions

The RTOG 8502 “QUAD shot” regimen using VMAT is effective for incurable HNC with highly reduced toxicity. Treatment with multiple cycles is recommended for better treatment response and/or survival.

## Data Availability

The data that support the findings of this study are available from the corresponding author, but restrictions apply to the availability of the data, which were used under license for the current study, and so are not publicly available. Data are, however, available from the authors upon reasonable request and with permission of the Institutional Research Ethics Board at Kumamoto University Hospital.

## Abbreviations

HNC: Head and neck cancer
RT: Radiotherapy
QOL: Quality of life
RTOG: Radiation Therapy Oncology Group
3D-CRT: Three-dimensional conformal radiotherapy
IMRT: Intensity-modulated radiotherapy
VMAT: Volumetric modulated arc therapy
OAR: Organs at risk
CT: Computed tomography
GTV: Gross tumor volume
MR: Magnetic resonance
FDG: [^18^F]-fluoro-2-deoxy-D-glucose
PET: Positron emission tomography
PTV: Planning target volume
CBCT: Cone-beam computed tomography
ART: Adaptive radiothrapy
OS: Overall survival
PFS: Progression-free survival
2D-RT: Two-dimensional radiotherapy
IGRT: Image-guided radiotherapy
